# The Molecular Relationship Between SDF4 and Thiol/Disulfide Homeostasis and Cardiac Injury Markers in Serum and Pericardial Fluid of Patients Undergoing Open-Heart Surgery

**DOI:** 10.3390/jcm14248942

**Published:** 2025-12-18

**Authors:** Murat Ziya Bağış, Ezhar Ersöz, İsmail Koyuncu, Kadir Eği, Bişar Amaç

**Affiliations:** 1 Department of Cardiovascular Surgery, Faculty of Medicine, Harran University, Sanliurfa 63300, Turkey; ziyabagis@hotmail.com (M.Z.B.);; 2 Department of Medical Biochemistry, Faculty of Medicine, Harran University, Sanliurfa 63300, Turkey; 3 Department of Medical Biochemistry, Faculty of Medicine, Gaziantep University, Gaziantep 27410, Turkey; 4 Vocational School of Health Services, Dialysis Program, Harran University, Sanliurfa 63300, Turkey; 5 Department of Perfusion, Faculty of Health Sciences, Harran University, Sanliurfa 63300, Turkey

**Keywords:** coronary artery disease, pericardial fluid, serum biomarkers, SDF4, thiol/disulfide balance

## Abstract

**Background/Objectives**: Various pathophysiological mechanisms play a role in the development of cardiovascular diseases (CVDs). There is a need for new biomarkers that can complement existing clinical findings, particularly in the early diagnosis and prognostic assessment of coronary artery disease (CAD) and that can also contribute to more effective management of the diagnosis and treatment process. Therefore, both blood and pericardial fluid samples can provide important diagnostic information. This study aims to investigate Stromal Cell-Derived Factor 4 (SDF4) levels and thiol/disulfide homeostasis in the blood and pericardial fluid of patients with established CAD undergoing open-heart surgery with cardiopulmonary bypass (CPB), in order to better characterize oxidative stress-related and redox-mediated pathophysiological processes associated with the development and progression of coronary heart disease. Comparisons with a healthy control group were performed to elucidate disease-related biochemical alterations rather than to propose these markers as diagnostic tools for CAD. **Methods**: In this study, intraoperatively collected venous blood and pericardial fluid samples from 45 patients undergoing on-pump coronary artery bypass grafting were analyzed. SDF4 levels were measured using enzyme-linked immunosorbent assay (ELISA), while thiol–disulfide homeostasis was assessed via spectrophotometric analysis. **Results**: The study revealed statistically significant differences in parameters such as SDF-4, native thiol, total thiol, disulfide, and disulfide/total thiol ratio among the control, patient serum, and pericardial fluid groups (*p* < 0.05). Notably, SDF-4 and disulfide levels were elevated, while thiol levels were reduced in the pericardial fluid group, suggesting increased oxidative stress and disrupted redox balance. Principal Component Analysis (PCA) and Variable Importance in Projection (VIP) analyses successfully demonstrated the discriminative power of these parameters among the groups. **Conclusions**: The increased SDF-4 levels and disturbances in the thiol–disulfide balance observed in this study indicate elevated oxidative stress and impaired cellular redox homeostasis in CAD. These findings suggest that SDF-4 and thiol–disulfide parameters may serve as important biochemical markers in the pathophysiology of CVD and hold potential as diagnostic and prognostic biomarkers.

## 1. Introduction

Cardiovascular diseases (CVD) remain among the leading causes of death worldwide [1]. During the diagnostic process, both blood and pericardial fluid (PF) are considered to provide valuable information. Pericardial fluid is a plasma ultrafiltrate originating from the cardiac interstitium and passing through the epicardial layer into the pericardial cavity [2].

The analysis of this biological fluid, which is located in closest proximity to the heart, may contribute to a better understanding of the pathophysiology of CVD. For example, significantly elevated levels of atrial natriuretic peptide (ANP) and brain natriuretic peptide (BNP) in the pericardial fluid of patients with heart failure have been reported. This increase suggests that these peptides may play pathophysiological roles through autocrine or paracrine mechanisms in heart failure [3].

Furthermore, oxidative stress has been shown to play a significant role in the pathogenesis of various CVD such as atherosclerosis, heart failure, cardiac arrhythmias, and ischemia–reperfusion injury [4]. Human life is sustained by aerobic metabolic processes that utilize oxygen, and during these processes, reactive oxygen species (ROS) are generated as byproducts. ROS are natural but potentially harmful products of cellular metabolism. Increased oxidative stress can lead to structural alterations in lipids, deoxyribonucleic acid (DNA), and proteins, thereby triggering cellular inflammation and causing programmed cell death [5]. In individuals with CVD, elucidating the molecular mechanisms underlying the condition is of great importance for the identification of novel biomarkers and the development of therapeutic agents aimed at disease prevention [6].

The oxidative effects generated by reactive oxygen species (ROS) are counteracted by the cells’ antioxidant defense systems, thereby mitigating oxidative stress and maintaining cellular homeostasis [7]. The regulation of redox couples within the cell occurs in a compartment-specific manner, particularly in organelles such as the mitochondria, endoplasmic reticulum, and nucleus [8]. Additionally, extracellular compartments provide an extra layer of protection by forming defensive barriers against external oxidants. Cysteine (Cys) and its disulfide form, cystine (CySS), constitute the primary low-molecular-weight thiol/disulfide pair in human plasma. The Cys/CySS pool functions as a central redox control point in biological signal transduction [9].

Stromal cell-derived factors (SDFs) comprise a family of proteins that includes SDF1 (CXCL12), SDF2, SDF2-like 1 (SDF2L1), SDF3, SDF4, and SDF5 and are primarily synthesized by stromal cells, particularly fibroblasts. The most extensively characterized member of this family is SDF1 (CXC motif chemokine 12; CXCL12), which plays crucial roles in key physiological and pathophysiological processes such as hematopoiesis, cell migration, and tumor metastasis. In contrast, the functions of SDF2, SDF3, SDF4, and SDF5 remain largely unclear. SDF4—also known as Cab45—is a member of the CREC protein family and contains six EF-hand motifs, which are calcium-binding domains. These structural features suggest that SDF4 may play an active role in cellular processes such as calcium signaling and protein trafficking within the Golgi apparatus [10].

This study aims to investigate Stromal Cell-Derived Factor 4 (SDF4) levels and thiol/disulfide homeostasis in the blood and pericardial fluid of patients with established coronary artery disease (CAD) undergoing open-heart surgery with cardiopulmonary bypass (CPB), in order to better characterize oxidative stress–related and redox-mediated pathophysiological processes associated with the development and progression of coronary heart disease. Comparisons with a healthy control group were performed to elucidate disease-related biochemical alterations rather than to propose these markers as diagnostic tools for CAD. This aims to obtain more comprehensive data on the biochemical and molecular properties of CAD.

## 2. Methods

In this study, approval was obtained from the institutions and the local ethics committee (Harran University Clinical Research Ethics Committee) (Date: 30 June 2025—Approval no: HRÜ/25.12.37). The study was conducted following the principles of the Declaration of Helsinki. The study was conducted with 45 patients who underwent coronary artery bypass surgery with CPB in the cardiovascular surgery operating theater of the relevant hospital and 45 healthy volunteers (control group). Patients and individuals in the control group were included in the study between 1 July 2025 and 30 September 2025.

All individuals in the patient group included in the study were patients diagnosed with isolated CAD who were scheduled to undergo surgery with CPB. Acute myocardial infarction, unstable angina, or additional cardiac pathologies were excluded from the study. The control group consisted of healthy individuals without any cardiovascular disease, systemic inflammatory condition, or chronic illness.

### 2.1. Collection of Blood and Pericardial Fluid During Cardiopulmonary Bypass

In open-heart surgeries, the temporary cessation of cardiac activity and the creation of a bloodless surgical field are critically important for ensuring a safe and effective procedure. To achieve this, CPB is utilized during the operation to maintain systemic circulation while the heart is temporarily arrested. In this study, venous blood and pericardial fluid samples were collected from patients diagnosed with CAD and scheduled for elective open-heart surgery, during routine surgical procedures. Following the standard CPB procedure and median sternotomy, the pericardial sac was carefully opened, and pericardial fluid samples were aspirated using a sterile syringe. The collected venous blood and pericardial fluid samples were transferred into sterile, anticoagulant-free tubes and immediately placed in an ice-filled container to preserve sample integrity. Subsequently, all samples were promptly centrifuged to separate them from cellular components. The resulting supernatant phase was transferred into RNase-free sterile tubes and stored at −80 °C for use in molecular analyses. After an adequate number of blood and pericardial fluid samples had been collected, analyses were performed to determine the levels of SDF4 and thiol–disulfide homeostasis in the patient samples. These analyses enabled the precise measurement of protein levels and gene expressions, providing significant data regarding the potential roles of SDF4 as a biomarker in CVDs such as CAD.

### 2.2. Quantification of SDF4 in Blood and Pericardial Fluid by ELISA

In this study, the levels of SDF4 in blood and pericardial fluid samples were measured using commercial enzyme-linked immunosorbent assay (ELISA) kits provided by Elabscience Biotechnology Co., Ltd. (Wuhan, Hubei, China; catalog number: E-EL-H2402).

In blood samples, quantification of SDF4 was performed using both ELISA and quantitative real-time polymerase chain reaction (qRT-PCR) techniques. The procedures were performed in accordance with the protocols provided by the manufacturer to ensure the accuracy and reliability of the biomarker measurements. In blood samples, ELISA offered high sensitivity in determining protein concentrations, while qRT-PCR provided a precise analysis of gene expression levels. The combined use of these complementary techniques strengthened the validity of the findings obtained in this study.

### 2.3. Measurement of Thiol–Disulfide Homeostasis in Blood and Pericardial Fluid

This recently developed method is based on the principle of thiol–disulfide homeostasis, which involves the measurement of thiol groups in plasma before and after exposure to oxidative stress. Using this technique, the levels of native thiol (–SH) and total thiol (–SH + –S–S) are measured directly. The disulfide (–S–S) level, another key component of this homeostasis, is calculated as half the difference between total thiol and native thiol levels. In this context, native thiol, total thiol, and disulfide (SS) levels were determined. In addition, ratios such as disulfide/native thiol (%SS/SH), disulfide/total thiol (%SS/Total Thiol), and native thiol/total thiol (%SH/Total Thiol) were calculated. Thus, the three main parameters that define thiol–disulfide balance—native thiol, disulfide, and total thiol—were comprehensively assessed [11].

### 2.4. Statistical Analysis

The statistical analyses of the study data were performed using SPSS (Statistical Package for the Social Sciences) version 27.0 (IBM Corp., Armonk, NY, USA) and MetaboAnalyst 6.0. Quantitative data were expressed as mean ± standard deviation (Mean ± SD). One-way analysis of variance (One-Way ANOVA) was used to compare the three groups (control, patient serum, and pericardial fluid). For variables showing a significant difference in ANOVA, Tukey’s post hoc test was applied to identify which groups differed from each other. To determine the primary discriminative variables, Variable Importance in Projection (VIP) scores were evaluated, and PLS-DA (Partial Least Squares Discriminant Analysis) was conducted. Additionally, to visualize the biochemical profile differences among the sample groups, two-dimensional and three-dimensional PCA (Principal Component Analysis) analyses were performed, along with biplot and heatmap visualizations. A *p*-value of <0.05 was considered statistically significant for all tests.

## 3. Results

### 3.1. Demographic Data

A total of 45 patients participated in the study, including 16 females and 29 males. The distribution of the groups according to body mass index (BMI) and as well as the mean age (Table 1), and the distribution of gender and smoking status (Table 2), are presented below. The observed difference in age between the two groups was found to be at the borderline of statistical significance (*p* = 0.05). This finding suggests that CAD tends to occur at older ages, which is consistent with the literature [12]. Regarding height, the difference between the groups reached statistical significance (*p* = 0.059), although it is not considered clinically meaningful. No statistically significant difference was found between the groups in terms of body weight (*p* = 0.29). When Body Mass Index (BMI) was calculated, no significant difference was observed between the groups. Regarding gender distribution, the patient and control groups also showed similar proportions (female: 35.6% vs. 42.2%; male: 64.4% vs. 57.8%), with no significant difference observed between the groups (*p* = 0.52). For smoking status, both groups exhibited similar rates (58% vs. 62%), and no significant difference was observed (*p* = 0.67). This indicates that smoking was equally distributed across the groups and is unlikely to act as a confounding variable influencing the other parameters of the study. The clinical and anamnestic characteristics of the study population are summarized in Table 2. In the patient group, hypertension was present in 62.2% of patients, diabetes mellitus in 37.8%, dyslipidemia in 66.7%, and a history of myocardial infarction in 31.1%. A positive family history of coronary artery disease was significantly more frequent in patients than in the control group (42.2% vs. 20.0%, *p* = 0.02).

### 3.2. Comparative Evaluation of SDF-4 and Thiol–Disulfide Homeostasis Parameters

**Figure 1:**
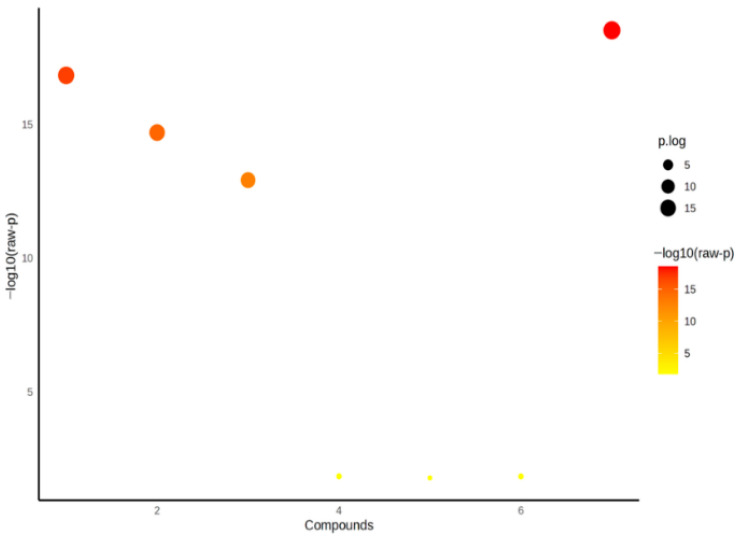
Visualization of SH/Total Thiol Ratio, SDF-4, and Native Thiol Levels Using a Bubble Chart.

Table 3 presents the statistical significance levels of various parameters among the control, patient serum, and pericardial fluid groups as evaluated by ANOVA. These findings, supported by bubble charts, reveal pronounced differences particularly in the SH/Total Thiol ratio, SDF-4, and Native Thiol levels (Figure 1). According to the post hoc analysis results, these three parameters demonstrated the most significant variation across groups (*p* < 0.001).

**Figure 2:**
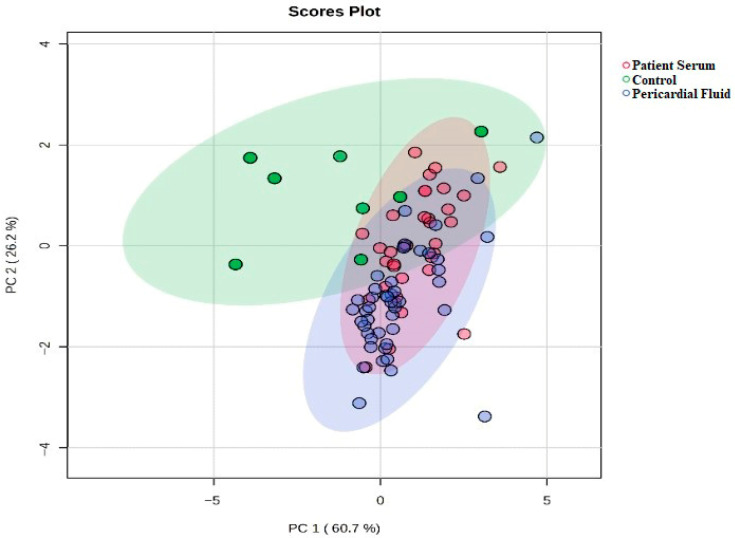
Two-Dimensional PCA Score Plot.

The two-dimensional PCA score plot visualizes the overall variation and similarities among the control, patient serum, and pericardial fluid groups. The clear separation of the groups on the plot indicates statistically significant differences in their biochemical profiles (Figure 2). In particular, the distinct positioning of the PS group suggests that it possesses a unique metabolic profile. These results demonstrate that the PCA effectively reflects intergroup separation and that the evaluated biomarkers have strong discriminative potential.

**Figure 3:**
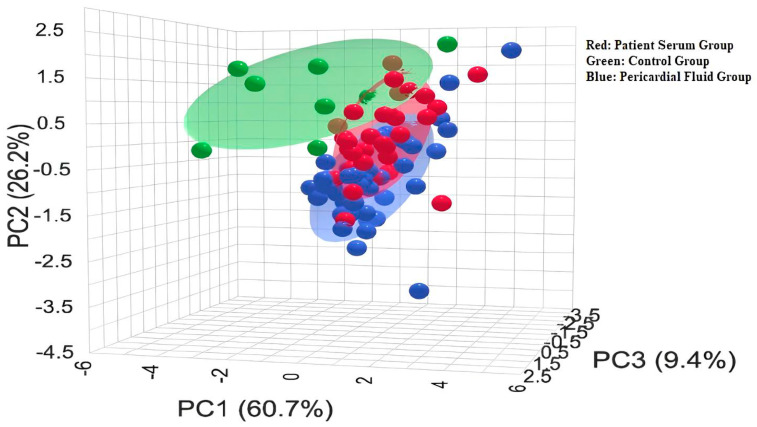
Three-Dimensional PCA Score Plot.

The three-dimensional PCA plot presents the distribution of the control (green), patient serum (red), and pericardial fluid (blue) groups based on their biochemical profiles (Figure 3). The principal components PC1, PC2, and PC3 explain 60.7%, 26.2%, and 9.4% of the variance, respectively, thus accounting for the majority of the dataset. The distinct positioning of patient serum and pericardial fluid samples compared to the control group indicates that these groups possess biochemically distinct profiles. Furthermore, the proximity between the patient serum and pericardial fluid groups suggests the presence of partial similarities between them.

**Figure 4:**
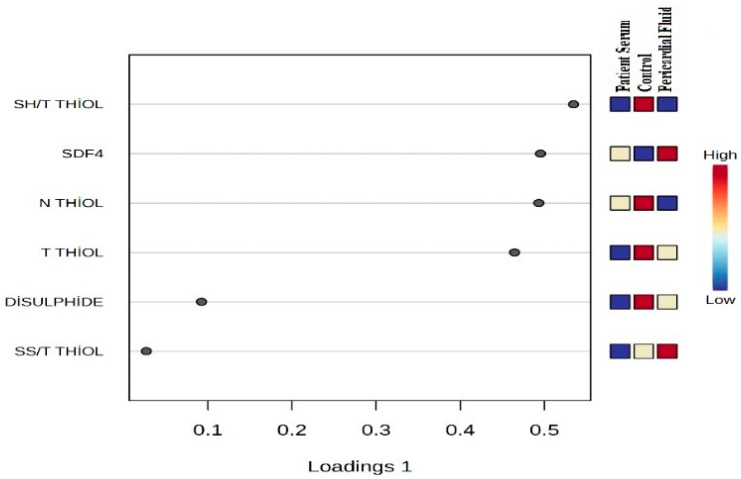
Variable Importance in Projection (VIP) Score Plot.

The VIP score plot demonstrates the discriminative power of thiol–disulfide balance parameters among the control, patient serum, and pericardial fluid (PS) groups. According to the PLS-DA analysis, the SS/Total Thiol ratio, SDF-4, and Native Thiol levels have the highest VIP scores and contribute most significantly to explaining the biochemical differences between the groups (Figure 4). These findings indicate that the mentioned parameters reflect disease-related biochemical alterations and may be considered potential biomarkers.

**Figure 5:**
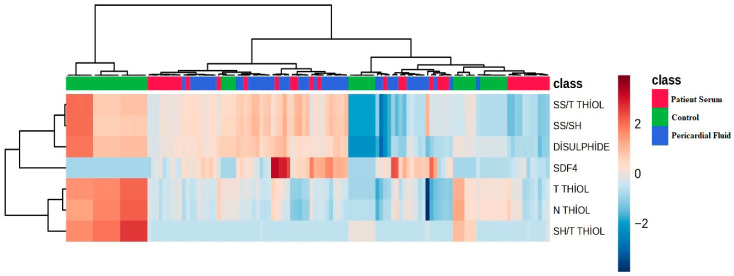
Heatmap Visualization.

The heatmap visually presents the comparative levels of thiol–disulfide balance parameters measured in the control, patient serum, and pericardial fluid (PS) groups. Color intensity represents the level of each variable within the respective group (Figure 5). A significant increase is observed in the SS/Total Thiol ratio, Disulfide, and SDF4 values in the patient groups compared to the control group, whereas a noticeable decrease is seen in Native Thiol and Total Thiol levels. This pattern indicates enhanced oxidative stress and disruption of redox homeostasis under pathological conditions.

**Figure 6:**
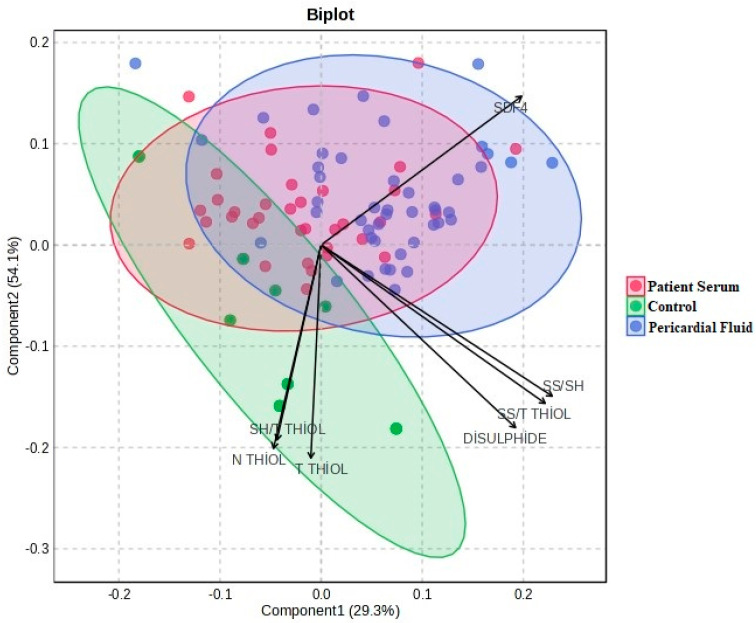
Biplot Graph.

The PCA-based biplot graph illustrates the distribution among groups and the contribution levels of each variable to this distribution. The orientation of the SS/Total Thiol, Disulfide, and SDF4 vectors toward the patient groups indicates that these variables are elevated in individuals with the disease. In contrast, Native Thiol and Total Thiol variables are positioned closer to the control group, suggesting higher levels in healthy individuals (Figure 6). The lengths of the vectors represent each parameter’s contribution to the variance, with variables like SS/Total Thiol and Disulfide playing a particularly decisive role in distinguishing between groups.

## 4. Discussion

Excessive production of reactive oxygen species (ROS) plays a significant role in the pathogenesis of various CVDs. Under normal physiological conditions, a delicate balance exists between ROS generation and antioxidant defense systems. However, when this balance is disrupted due to elevated ROS levels, oxidative stress increases. Heightened oxidative stress can induce structural and functional alterations in cardiomyocytes, paving the way for serious cardiac dysfunctions such as heart failure [13]. Thiols (RSH) react with oxidants to form both reversible thiol oxidation products (RTOP) and irreversible thiol oxidation products (ITOP). These products include disulfide, sulfenic acid, sulfinic acid, and sulfonic acid. RTOPs, such as disulfide bonds, are dynamic oxidation products that can be reduced back to thiol groups through endogenous antioxidant mechanisms. This reversible transformation plays a crucial role in maintaining thiol–disulfide homeostasis [14].

The use of thiol–disulfide homeostasis in CVDs has gained considerable attention in recent years, fueled by a growing body of research. This system is recognized as a crucial biomarker of oxidative stress and provides valuable insights into the pathogenesis, prognosis, and treatment response of various CVDs. Kundi et al. demonstrated significantly elevated disulfide levels in patients with acute coronary syndrome [15]. Previous studies have also reported increased lipid peroxidation following thrombolytic therapy in patients who experienced myocardial infarction (MI) [16], and have shown that disrupted thiol–disulfide homeostasis plays a role in the pathogenesis of CVDs [17]. In another study by Burns et al., it was revealed that glyceraldehyde-3-phosphate dehydrogenase (GAPDH) undergoes glutathionylation in human aortic endothelial cells, leading to decreased GAPDH activity. This alteration contributes to increased disulfide stress, impairs glycolysis, and ultimately promotes apoptosis [18].

In our study, significant alterations were observed in thiol–disulfide balance parameters in both pericardial fluid and patient serum. Notably, total and native thiol levels were markedly reduced in the patient groups compared to the control group, while disulfide levels and the disulfide/total thiol ratio were found to be elevated. These findings support the notion that oxidative stress plays a critical role in CVDs. Thiol compounds, which contain sulfhydryl groups, serve as essential antioxidants in the body by reacting with free radicals and preventing cellular damage. The increase in disulfide levels reflects the oxidation of thiols under oxidative conditions, indicating a disruption of redox homeostasis. The elevated disulfide concentrations observed in our study suggest enhanced oxidative stress and weakened antioxidant defense mechanisms in patients. This imbalance in redox status likely contributes to CVD pathogenesis. Furthermore, disturbances in thiol–disulfide homeostasis may directly impact myocardial cell function, potentially altering intracellular signaling pathways and triggering inflammation and apoptosis. In this context, the assessment of thiol–disulfide parameters holds significant promise as potential biomarkers in the diagnosis and prognosis of CVD.

Some studies have demonstrated that oxidative stress not only causes damage at the cellular level but also accelerates processes such as the development of CAD and atherosclerotic plaque formation [19]. In a study by Vukasović and colleagues, it was shown that malondialdehyde levels in both plasma and pericardial fluid could serve as early indicators of ventricular dysfunction. This finding supports the notion that pericardial fluid is not merely a passive ultrafiltrate but rather a dynamic biological environment capable of directly reflecting cardiac pathophysiological processes [3].

Coronary artery disease is one of the leading conditions posing a serious public health threat, with its global prevalence steadily increasing. Despite significant advances in the treatment of CVDs, they continue to rank among the top causes of global mortality [1]. In the diagnosis of CVDs, not only blood and cardiac tissue but also pericardial fluid is considered a valuable biological sample. Although not directly a part of the heart, the anatomical proximity of pericardial fluid endows it with the potential to carry unique and valuable insights into the pathophysiological state of the cardiovascular system. With the advancement of techniques overcoming the limitations of invasive sampling methods such as cardiac surgery or pericardiocentesis, the analysis of pericardial fluid content has emerged as a notable area of interest in cardiovascular diagnostics, disease monitoring, and pathogenesis research [20]. Alongside efforts to elucidate the molecular mechanisms of the disease, effective early diagnostic and therapeutic approaches play a critical role in managing disease severity and preventing the emergence and progression of comorbid conditions [21]. Iskandar et al. and Silva et al. [22,23] compared pericardial fluid levels across various cardiovascular pathologies. These studies reported a significant increase in pericardial fluid levels among patients with symptoms of congestive heart failure, while no notable differences were observed in individuals with ischemic heart disease or stable valvular disease. These findings suggest that pericardial fluid can be considered a biological medium that reflects the functional status of the heart.

The pericardial cavity has the potential to act as a homeostatic niche that reflects both the physiological and pathophysiological status of the heart. However, our current understanding of pericardial fluid remains largely limited to routine hematological, biochemical, and cytological analyses performed only when clinical necessity allows access to the pericardial space. Consequently, there is a pressing need for more comprehensive and targeted investigations into the potential biomarker content of pericardial fluid. Butts et al. [24] demonstrated that levels of certain proinflammatory markers remain elevated in thoracic fluid following cardiac surgery, suggesting that this is a consequence of tissue injury and the associated inflammatory response induced by the surgical procedure. On the other hand, recent studies conducted on zebrafish have revealed that SDF4 plays a significant role in the process of vasculogenesis. Vasculogenesis and angiogenesis are two fundamental biological mechanisms involved in the formation of new blood vessels. SDF4 proteins have been shown to localize in endothelial cells, with their expression increasing under hypoxic conditions. These findings provide important insights into the angiogenic properties of SDF4 and its potential contribution to neovascularization [25].

The patient population included in this study exhibited a high prevalence of established cardiovascular risk factors, such as hypertension, diabetes mellitus, dyslipidemia, and a history of myocardial infarction, consistent with a typical coronary artery disease phenotype. In addition, a positive family history of coronary artery disease was significantly more frequent in patients compared with healthy controls. These clinical characteristics are known to be associated with increased oxidative stress, chronic inflammation, and endothelial dysfunction, which may have contributed to the observed alterations in SDF4 levels and thiol/disulfide homeostasis. Therefore, the biochemical findings of the present study should be interpreted in the context of the underlying clinical risk profile of the patient population.

In our study, SDF4 levels were found to be significantly elevated in both patient serum and pericardial fluid compared to the control group. SDF4 is known as a calcium-binding protein located in the Golgi apparatus, playing a role in intracellular protein transport and calcium signaling. However, its functions in CVDs have not yet been fully elucidated. The observed increase in SDF4 suggests that it may be associated with cellular stress and inflammatory responses involved in the pathogenesis of CVDs. Given the critical importance of calcium signaling in cardiomyocyte function and vascular homeostasis, the possibility that SDF4 could serve as a potential biomarker or pathophysiological mediator in these diseases is reinforced. Additionally, elevated SDF4 levels are thought to have a regulatory role in processes such as inflammation, cell migration, and tissue repair. Additionally, in our study, important clinical variables that may affect redox biomarkers, such as diabetes, hypertension, dyslipidemia, statin or ACE inhibitor use, which may affect oxidative stress biomarkers, may be present in the disease group. In contrast, as the control group consists of healthy individuals, these variables are not naturally observed. Although this situation limits the possibility of achieving “absolute clinical matching” between the groups, this approach is considered methodologically acceptable since the primary aim of the study is to evaluate disease-related biochemical changes.

### Limitations

This study has several limitations. First, it was conducted at a single center with a relatively limited number of patients, which may restrict the generalizability of the findings to broader patient populations. Additionally, samples were collected at a single time point during surgery, preventing the assessment of dynamic changes in biomarker levels over time. This is particularly important for understanding the postoperative progression of inflammatory and oxidative stress responses. Moreover, since pericardial fluid sampling is influenced by physiological changes induced by surgical intervention, the measured levels of SDF4 and thiol/disulfide parameters may not fully reflect the baseline cardiac status.

The fact that pericardial fluid samples were obtained during surgery constitutes one of the important methodological limitations of this study. Surgical intervention can affect biomarker levels by causing a marked inflammatory response and oxidative stress in cardiac tissues and surrounding anatomical structures. In particular, cardiopulmonary manipulation, tissue ischemia–reperfusion processes, and the effects of anesthetic agents on the biochemical response may create surgery-specific alterations in pericardial fluid composition. Therefore, some of the increases in biomarkers detected in pericardial fluid may reflect the acute effects of surgical stress in addition to underlying cardiovascular pathology. Furthermore, as hemodynamic changes and the release of local inflammatory mediators during surgery can cause significant short-term fluctuations in the biochemical profile of pericardial fluid, this should be taken into account when interpreting the results. Since it is technically and ethically impossible to completely control the surgical stress response, it should be borne in mind that some of the measured values may be the result of systemic and local biological responses developing during the operation rather than the primary pathophysiological processes of the disease.

Another limitation is the lack of correlation between the biomarker levels and long-term clinical outcomes such as mortality, morbidity, or hospital readmissions, which restricts the ability to evaluate the prognostic value of these parameters. Therefore, to strengthen and validate these findings, future research should involve larger, multi-center, prospective studies that incorporate longitudinal sampling to monitor temporal changes in biomarker levels. Furthermore, in comparing serum/plasma biomarker levels with pericardial fluid, the structural and functional differences between the two biological compartments represent a significant limitation. As PCF sampling was not possible in the control group, it is not possible to fully distinguish whether the observed differences are due to disease or anatomical compartment. This situation has been noted as a methodological limitation that should be considered when interpreting our results.

## 5. Conclusions

In this study, it was demonstrated that SDF4 levels were significantly elevated and the thiol–disulfide balance was markedly disrupted in serum and pericardial fluid samples obtained from patients undergoing open-heart surgery due to CAD. The increase in SDF4 suggests its potential role in key pathological processes of CVDs, including inflammation, cellular stress responses, and tissue remodeling. Similarly, the rise in disulfide levels alongside the reduction in thiol levels indicates enhanced oxidative stress and weakened antioxidant defense. These findings suggest that both SDF4 and the thiol–disulfide balance may serve as potential biochemical and molecular biomarkers in understanding the pathophysiology of CAD. Further investigation of these parameters in future clinical studies with larger patient cohorts may provide valuable insights for the early diagnosis, prognosis assessment, and development of novel therapeutic targets in CVD management.

## Figures and Tables

**Table 1:** Comparison of Age and Body Mass Index Between the Patient and Control Groups.

	Patient			Control			
	Min	Max	Mean ± SD	Min	Max	Mean ± SD	*p* Value
Age (years)	29	76	56.92 ± 10.12	30	55	41.27 ± 9.85	0.05
BMI (kg/m^2^)			28.41 ± 5.11			26.92 ± 4.24	0.134

Body Mass Index: BMI.

**Table 2:** Clinical and Anamnestic Characteristics of Patient and Control Groups.

		Patient Group		Control Group		
		%	n	%	n	*p* Value
Gender	Female	35.56	16	42.22	19	0.52
	Male	64.44	29	57.78	26	
Smoking	Yes	58	26	62	28	0.67
	No	42	19	38	17	
Hypertension		62.2	28			
Diabetes mellitus		37.8	17			
Dyslipidemia		66.7	30			
Family history of CAD		42.2	19	20	9	**0.02**
Previous myocardial infarction		31.1	14			
Total		100	45	100	45	

CAD: Coronary Artery Disease.

**Table 3:** Comparison of SDF-4 and Thiol–Disulfide Homeostasis Parameters Among Control, Patient Serum, and Pericardial Fluid Groups.

	Control (A)			Patient Serum (B)			Pericardial Fluid (C)				
	Min	Max	Mean ± SD	Min	Max	Mean ± SD	Min	Max	Mean ± SD	*p* ^a^	Post Hoc ^b^
SDF-4	40.54	114.47	70.92 ± 23.17	84.00	358.23	127.99 ± 54.72	82.65	364.38	162.46 ± 63.08	0.001 **	A-B, B-C
NATİVE THİOL (SH)	261.60	469.50	359.4 ± 52.46	199.50	461.60	309.44 ± 57.54	75.87	444.46	286.75 ± 58.69	0.001 **	A-B, B-C
TOTAL THİOL	273.50	516.19	401.6 ± 59.49	224.00	493.90	346.28 ± 60.83	92.94	474.02	332.83 ± 63.61	0.001 **	A-B, B-C
DİSULPHİDE (SS)	5.95	39.12	21.1 ± 9.36	6.98	33.45	18.42 ± 5.97	4.56	35.03	23.04 ± 7.33	0.01 *	A-B, B-C
% SS/SH	2.27	13.38	6.43 ± 3.16	2.37	11.46	6.1 ± 2.07	1.78	12.55	8.24 ± 2.66	0.06	
% SS/TOTAL THİOL	2.18	10.93	5.63 ± 2.45	2.26	9.33	5.38 ± 1.63	1.72	10.03	6.99 ± 2.02	0.04 *	A-B, B-C
% SH/TOTAL THİOL	81.65	113.92	97.72 ± 7.29	81.35	95.48	89.25 ± 3.26	79.94	96.57	86.03 ± 4.05	0.001 **	A-B, B-C

^a^ Anova Test, *p* < 0.001 **, *p* < 0.05 *, ^b^ Tukey Test.

## Data Availability

The original contributions presented in this study are included in the article. Further inquiries can be directed to the corresponding author.
